# Administration of Intravenous Iron Formulations Induces Complement Activation *in-vivo*

**DOI:** 10.3389/fimmu.2019.01885

**Published:** 2019-08-21

**Authors:** Bernardo Faria, Mariana Gaya da Costa, Felix Poppelaars, Casper F. M. Franssen, Manuel Pestana, Stefan P. Berger, Mohamed R. Daha, Carlo A. J. M. Gaillard, Marc A. Seelen

**Affiliations:** ^1^Division of Nephrology, Department of Internal Medicine, University Medical Center Groningen, University of Groningen, Groningen, Netherlands; ^2^Department of Nephrology, Hospital de Braga, Braga, Portugal; ^3^Nephrology and Infectious Disease R&D Group, INEB, I3S, University of Porto, Porto, Portugal; ^4^Department of Nephrology, Leiden University Medical Center, University of Leiden, Leiden, Netherlands; ^5^Division of Internal Medicine and Dermatology, University Medical Center Utrecht, University of Utrecht, Utrecht, Netherlands

**Keywords:** kidney, complement, hemodialysis, iron, anemia and kidney disease

## Abstract

**Background:** Intravenous (IV) iron is widely used to treat anemia in chronic kidney disease patients. Previously, iron formulations were shown to induce immune activation *in-vitro*. The current study aimed to investigate the effect of IV iron on complement activation *in-vivo*, and whether this subsequently induces inflammation and/or oxidative stress.

**Methods:** Two distinct patient groups were included: 51 non-dialysis and 32 dialysis patients. The non-dialysis group received iron sucrose or ferric carboxymaltose, based on physicians' choice. Plasma samples were collected prior to and 1 h after completion of IV iron infusion. The dialysis group received iron sucrose exclusively. Plasma samples were collected at the start and end of two consecutive hemodialysis sessions, one with and one without IV iron. Finally, plasma levels of MBL, C1q, properdin, factor D, sC5b-9, MPO, PTX3 were assessed by ELISA.

**Results:** In the non-dialysis group, sC5b-9 levels significantly increased after IV iron by 32%, while levels of factor D and MBL significantly dropped. Subgroup analysis demonstrated that iron sucrose induced complement activation whereas ferric carboxymaltose did not. In the dialysis group, levels of sC5b-9 significantly increased by 46% during the dialysis session with IV iron, while factor D levels significantly fell. Furthermore, the relative decrease in factor D by IV iron correlated significantly with the relative increase in sC5b-9 by IV iron. MPO levels rose significantly during the dialysis session with IV iron, but not in the session without iron. Moreover, the relative increase in MPO and sC5b-9 by IV iron correlated significantly. PTX3 levels were not affected by IV iron.

**Conclusions:** Iron sucrose but not ferric carboxymaltose, results in complement activation possibly via the lectin and alternative pathway partially mediating oxidative stress but not inflammation.

## Introduction

Intravenous (IV) iron drugs are a mainstay in the management of anemia (1). A growing number of chronic kidney disease (CKD) patients receive IV iron (1, 2). In the past, safety concerns existed, since IV iron has been linked to iron overload, increased oxidative stress, cardiovascular risk, infection risk, and hypersensitivity reactions (1, 3). Recently, a multicenter open-label clinical trial with over 2,000 hemodialysis (HD) patients demonstrated that a high-dose IV iron sucrose regimen administered pro-actively resulted in lower cardiovascular events (29 vs. 32%) when compared to a low-dose IV iron sucrose regimen, while no difference in infection and mortality was seen (4). However, some questions persist about the long-term safety of IV iron, the comparison of different iron formulations and the clinical outcome in non-dialysis patients. Therefore, a better understanding of the IV iron (side) effects remains a relevant subject of research.

Previously, hypersensitivity reactions induced by IV iron have been proposed to arise from complement activation-related pseudo-allergy (CARPA) (5). Complement activation can be initiated via three different pathways: the classical pathway (CP), the lectin pathway (LP), and the alternative pathway (AP). All the pathways converge at the level of C3 and ultimately results in generation of C5b-9, the membrane attack complex (MAC) (6). Hypersensitivity reactions are extremely rare, making it difficult to establish a cohort large enough to investigate the CARPA hypothesis (7). Nevertheless, the concept of complement activation by IV iron warrants further investigation. A few studies have previously shown the ability of IV iron formulations to activate complement *in-vitro* (8, 9). For instance, low-molecular weight iron dextran, ferric gluconate, ferric carboxymaltose, and iron sucrose were all shown to activate complement. However, the paradoxical results found in these studies, emphasize the need for further research.

The current study aimed to investigate the effect of IV iron on complement activation *in-vivo*. In addition, we explored if a potential IV iron induced complement activation could lead to oxidative stress and inflammation. Therefore, complement activation was assessed through the measurement of sC5b-9 levels and components of different pathways, namely C1q from the CP, MBL from the LP and factor D and properdin from the AP. Also, inflammation and oxidative stress were studied through the measurement of myeloperoxidase (MPO) and pentraxin-3 (PTX-3). All these measurements were performed in distinct groups receiving IV iron treatment.

## Materials and Methods

### Study Design and Population

Patients were recruited from the day clinic and the dialysis unit of Hospital de Braga, Braga, Portugal. The first group included non-dialysis patients (CKD and non-CKD), receiving iron sucrose (Venofer®, Vifor Pharma) or ferric carboxymaltose (Ferinject®, Vifor Pharma) and the second group consisted of dialysis patients receiving only iron sucrose. Adult patients (≥18 years) receiving IV iron and who gave informed consent were eligible for the study. Exclusion criteria were administration of iron in the previous 3 months for the first group whereas signs of active inflammation was exclusion criteria for both groups.

In the non-dialysis group, the drug type and the dose of IV iron was based on the decision of the treating physician according to local clinical guidelines and protocols. Iron was infused with a slow infusion rate and complete drug administration occurred in 30 min for 100 mg of iron sucrose and 500 mg of ferric carboxymaltose, whereas higher doses of both drugs were infused in 1 h. Plasma EDTA samples were collected prior to and 1 h after completion of the iron infusion. During this period, patients were observed for allergic symptoms and signs, including itching, arthralgias, hypotension, tachycardia, respiratory symptoms, thoracic pain, edema, and/or rash.

In the dialysis group, patients received online-hemodiafiltration three times per week for 4 h using high flux polysulfone dialyzers (Fx80, Helixone, Fresenius Medical Care, St. Wendel, Germany). Two patients were dialyzed with cellulose triacetate filters (CT190G, Baxter, McGaw Park, IL, USA). Patients received iron sucrose starting 3 h after the start of the dialysis session through a slow IV injection over 2 min via the venous limb of the circuit. The treating physician decided the dose of the IV iron formulation. Samples were collected during two consecutive sessions, one with IV iron administration and one without. During each dialysis session, plasma EDTA samples were collected prior to dialysis (pre-dialysis) and after 4 h, at the end of the session (post-dialysis).

All blood samples were centrifuged within 30 min of collection at 3500 rpm for 15 min. Next, the samples were stored in aliquots at −80°C until the measurement of the different laboratory parameters. Prior to the assay, samples were thawed and re-centrifuged.

### Laboratory Procedures

Levels of sC5b-9 levels,C1q, MBL, factor D, and properdin were quantified in plasma EDTA samples by in-house sandwich ELISA as previously described (8, 10). In brief, plates were coated overnight at 4°C with the respective antibody in coating buffer (100 mM Na_2_CO_3_/NaHCO_3_, pH 9.6). After coating, plates were blocked using 1% BSA/PBS. Next, plasma samples were diluted into the appropriate buffer solution and incubated in the coated wells. The protein of choice was then detected using the DIG- or biotin-conjugated coating antibody followed by detection with streptavidin-poly-horseradish peroxidase or HRP-conjugated sheep anti-Dig Antibodies (Fab fragments, Roche, Mannheim, Germany). After each step the plate was washed in PBS Tween-20 (0.05%) and all incubation steps were done for 1 h at 37°C. For visualization 3,3′,5,5″-Tetramethylbenzidine (TMB) or 2,2′-azino-bis(3-ethylbenzothiazoline-6-sulphonic acid) (ABTS) was added and the optical density was measured at 450 or 415 nm, respectively. For the C1q and properdin ELISA, in house antibodies were used. For the other ELISAs anti-MBL 3e7 antibody (Hycult, Uden, The Netherlands), anti-factor D 18241 antibody (R&D systems, Wiesbaden-Norderstedt, Germany) or anti-C5b-9 AE11 antibody (DAKO, Copenhagen, Denmark) was used. The capture antibody for the sC5b-9 ELISA was different from the coating antibody and instead a goat anti-human C5 antibody was used (Sigma–Aldrich, St. Louis, MO, USA). MPO and PTX3 were measured using commercial ELISA kits according to the manufacturers' instructions (Hycult Biotech, Uden, The Netherlands).

### Statistics

Statistical analysis was performed using IBM SPSS 23.0 (IBM Corporation, Chicago, IL, USA). Laboratory measurements are shown as mean ± standard error of the mean (SEM). Comparisons between samples were made by paired sample *t*-test. Correlations were assessed using Pearson correlation coefficient (r). *P*-values <0.05 were considered to be statistically significant. The ratios used for the analyses were calculated per patient by dividing the pre-dialysis level by the post-dialysis level in both sessions. Subsequently the relative increase was calculated dividing the ratio of the session with iron, by the session without.

### Ethics

This study was performed in accordance to the Declaration of Helsinki and was approved by the Medical Ethical Committee from Hospital Braga. All participants signed informed written consent.

## Results

### Intravenous Infusion of Iron Sucrose Leads to Complement Activation in Non-dialysis Patients

The effect of IV iron on the complement system was first assessed in the non-dialysis group. This consisted of 51 patients with a median age of 64 years of which 63% were female (Table 1). Fifteen had non-dialysis CKD (ND-CKD) and 36 were non-CKD patients with anemia due to gastrointestinal or gynecological origin. Seventeen patients received ferric carboxymaltose at doses of 500 mg (*n* = 3) or 1,000 mg (*n* = 14), while 34 received iron sucrose at doses of 100 mg (*n* = 13), 200 mg (*n* = 20) or 300 mg (*n* = 1). No hypersensitivity reactions were observed.

**Table 1:** Baseline characteristics of the non-dialysis and dialysis patients.

	**Non-dialysis (*n* = 51)**	**Dialysis (*n* = 32)**
**Age, years**	64.3 ± 19.8	69.4 ± 15.7
**Female gender**, ***n*** **(%)**	32 (63)	9 (28)
**Weight, kg**	69.3 ± 13.6	71.7 ± 10.9
**BMI, kg/m**^**2**^	28.5 ± 4.9	26.5 ± 4.2
**Diabetes**, ***n*** **(%)**	13 (25)	17 (53)
**Anemia Etiology*****, n*** **(%)**		
Gastro-intestinal	27 (53)	0
Gynecological	9 (18)	0
Kidney Disease	15 (29)	32 (100)
Mixed^*^	8 (16)	4 (12)
**Stages of CKD (KDIGO)**, ***n*** **(%)**		
III	6 (40)	0
IV	6 (40)	0
V	3 (20)	32 (100)
**Hemoglobin, g/dL**	9.7 ± 2.1	11.4 ± 1.2
**Serum ferritin concentration, ng/mL**	48.1 ± 87.4	357 ± 188
**Transferrin saturation, %**	10.8 ± 8.3	30.9 ± 19.4
**Type of IV Iron**, ***n*** **(%)**		
Iron Sucrose	34 (67)	32 (100)
20 mg	0	4 (12)
50 mg	0	15 (47)
100 mg	13 (25)	13 (41)
200 mg	20 (39)	0
300 mg	1 (2)	0
Ferric Carboximaltose	17 (33)	0
500 mg	3 (6)	
1,000 mg	14 (27)	
**Other anemia treatments**, ***n*** **(%)**		
Oral Iron supplementation	18 (35)	0
Erythropoiesis stimulating agents	6 (12)	30 (94)

*BMI, body mass index; CKD, Chronic Kidney Disease; KDIGO, Kidney Disease Improving Global Outcomes; IV, intravenous*.

*All patients were Caucasians and data is expressed as means ± standard error of the mean or absolute numbers (percentage)*.

* *Involving more than one anemia etiology*.

**Figure 1:**
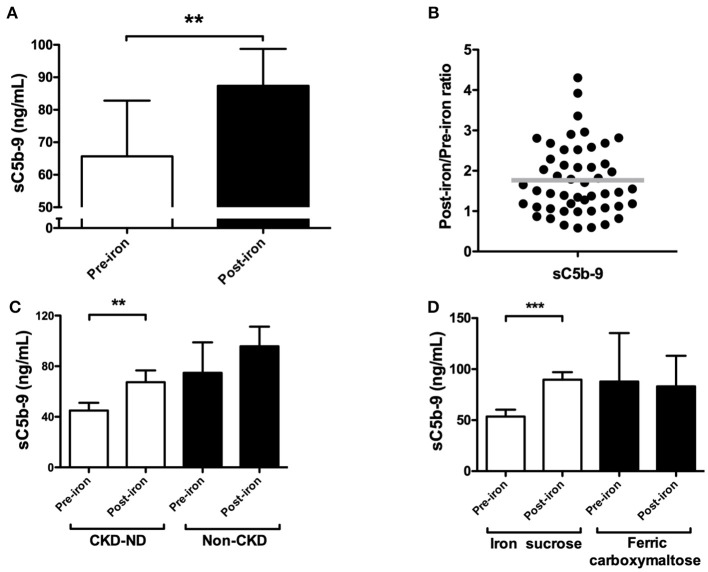
The effect of IV iron on complement activation, as assessed by soluble C5b-9 in non-dialysis patients. **(A)** The plasma levels of soluble C5b-9 (sC5b-9) were determined in 51 patients prior to and 1 h after completion of iron infusion. **(B)** The ratio of sC5b-9 was calculated per patient by dividing the pre-iron level by the post-iron level. Horizontal lines indicate the mean. A post/pre-ratio higher than 1, indicates an increase in concentration by iron. Subgroup analysis of plasma sC5b-9 levels in **(C)** chronic kidney disease (CKD) patients (*n* = 15) vs. non-CKD patients (*n* = 36). **(D)** In patients receiving ferric carboxymaltose (*n* = 17) vs. iron sucrose (*n* = 34). Data are presented as mean and SEM **(A,C,D)**. Paired sample t-test was used to compare values before and after iron infusion. P-values <0.05 were considered to be statistically significant (^**^P < 0.01, ^***^P < 0.001).

Overall, IV iron administration resulted in significant higher levels of sC5b-9 compared to baseline (*P* = 0.007; Figure 1A). The mean baseline values were 65.9 ± 17.1 ng/mL and rose to 87.3 ± 11.3 ng/mL after infusion, representing an average increase of 32% in sC5b-9 levels by IV iron. Nevertheless, the magnitude of the changes in levels of sC5b-9 by iron infusion varied, with 84% of patients showing complement activation by IV iron (Figure 1B). Next, we performed subgroup analyses of ND-CKD vs. non-CKD. In the ND-CKD group, levels of sC5b-9 were significantly increased by iron treatment (*P* = 0.007, Figure 1C), whereas in the non-CKD there was a trend for higher levels of sC5b-9 by IV iron (*P* = 0.05). Furthermore, infusion of iron sucrose was compared to ferric carboxymaltose. IV infusion with iron sucrose resulted in a significant rise in sC5b-9 levels (*P* < 0.001; Figure 1D). Baseline levels of sC5b-9 were 53.6 ± 6.6 ng/mL and after IV iron increased to 89.7 ± 7.1 ng/mL, showing an increase of 67%. In contrast, sC5b-9 levels were not significantly altered by iron infusion with ferric carboxymaltose (*P* = 0.76; Figure 1D). Of note, baseline levels were not statistically different between patients receiving iron sucrose compared to ferric carboxymaltose.

**Figure 2:**
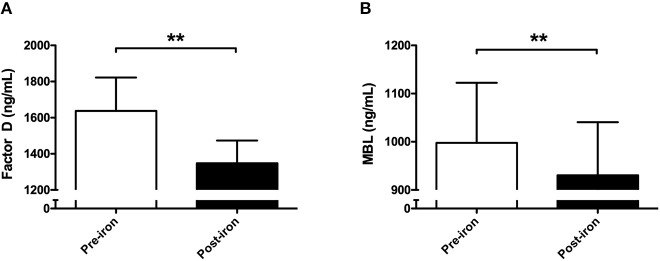
Consumption of mannose-binding lectin and Factor D following intravenous iron in non-dialysis patients. In 51 non-dialysis patients plasma levels of **(A)** factor D and **(B)** mannose-binding lectin (MBL) were determined prior to and 1 h after completion of iron infusion. Data are presented as mean and SEM. Paired sample *t*-test was used to compare values before and after iron infusion. P-values <0.05 were considered to be statistically significant (^**^P < 0.01).

To explore the effect of IV iron on complement components, C1q, properdin, factor D, and MBL were measured. Iron infusion was associated with a significant fall in the levels of factor D (*P* = 0.007; Figure 2A) and MBL compared to baseline (*P* = 0.006; Figure 2B), whereas properdin and C1q levels were not significantly altered after IV iron administration (Table 2).

**Table 2:** The effect of intravenous iron on C1q and properin in non-dialysis patients and the correlation with soluble C5b-9.

				**sC5b-9**					
	**Pre-iron**	**Post-iron**	P^*^	***R***	***P^#^***
**C1q** (μg/mL)	69.4 ± 4.5	64.9 ± 4.4	0.20	0.03	0.8
**Properdin** (μg/mL)	10.1 ± 0.8	8.8 ± 0.5	0.06	−0.14	0.3

Values are expressed as mean ± standard error of the mean. P^*^ indicates P-values for difference between samples pre and post-iron tested by paired t-test. R indicates Pearson correlation coefficient between Post-iron/Pre-iron ratio between the complement component and sC5b-9, and the corresponding P-value is shown by P^#^.

### Iron Infusion Has No Effect on Oxidative Stress or Inflammation in Non-dialysis Patients

**Figure 3:**
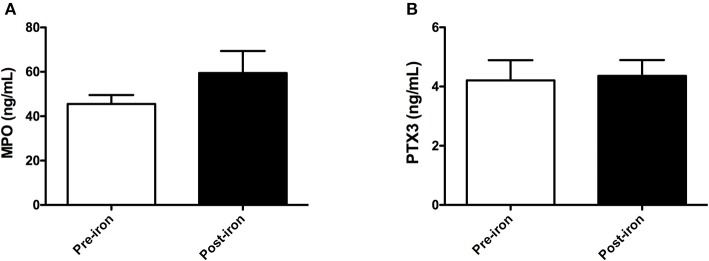
Pentraxin-3 and myeloperoxidase are not altered by intravenous iron in non-dialysis patients. In 51 non-dialysis patients, plasma levels of **(A)** myloperoxidase (MPO) and **(B)** pentraxin-3 (PTX3) were determined prior to and 1 h after completion of iron infusion. Data are presented as mean and SEM. Paired sample t-test was used to compare values before and after iron infusion. *P*-values <0.05 were considered to be statistically significant.

Subsequently, we determined the effect of IV iron on oxidative stress and inflammation by measuring levels of MPO and PTX3, respectively. Iron infusion resulted in a 33% increase of MPO levels, yet this rise was not statistically significant (*P* = 0.17; Figure 3A). PTX3 levels were unaffected by iron infusion, showing similar levels before and after iron infusion (*P* = 0.68; Figure 3B). In subgroup analysis, infusion of iron sucrose led to a non-significantly increase of MPO levels by 41% (*P* = 0.18), while PTX3 levels were not affected. The use of ferric carboxymaltose did not significantly affect MPO or PTX3 levels (data now shown).

### Intravenous Infusion of Iron Sucrose Leads to Complement Activation in Hemodialysis Patients

**Figure 4:**
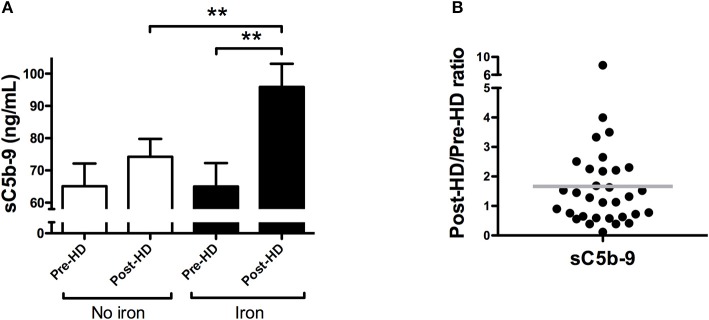
Iron sucrose results in complement activation in dialysis patients. **(A)** Plasma levels of soluble C5b-9 (sC5b-9) were determined in 32 hemodialysis patients during two consecutive dialysis sessions, one with intravenous iron (Iron), and one without (No iron). During each dialysis session, sC5b-9 levels were determined at baseline and after 4 h. Data are presented as mean and SEM. **(B)** The relative change in sC5b-9 was calculated per patient by dividing the pre-dialysis level by the post-dialysis level in both sessions and subsequently diving the session with iron, by the session without. Horizontal lines indicate the mean. A post/pre-ratio higher than one, indicates an increase in concentration of sC5b-9 by iron sucrose. The paired sample *t*-test was used to compare values before and after iron infusion. *P*-values <0.05 were considered to be statistically significant (^**^P < 0.01).

Next, we analyzed the effect of iron sucrose on complement activation in 32 dialysis patients, at doses of 100 mg (*n* = 13), 50 mg (*n* = 15), and 20 mg (*n* = 4). The mean age was 69 years and 72% were male (Table 1). During the study, no hypersensitivity reactions were observed in the dialysis patients. In the dialysis session without IV iron, sC5b-9 levels were not significantly increased after dialysis (*P* = 0.27; Figure 4A). However, IV iron during dialysis resulted in a significant rise of levels of sC5b-9 compared to baseline (*P* = 0.001) as well as compared to the end of the dialysis session without iron (*P* = 0.002; Figure 4A). IV iron resulted in an increase of 46% in sC5b-9 levels, compared to a 14% increase in the dialysis without IV iron. Of note, the magnitude of the change in sC5b-9 levels by iron infusion varied, with 60% of the dialysis patients showing complement activation by IV iron (Figure 4B).

**Figure 5:**
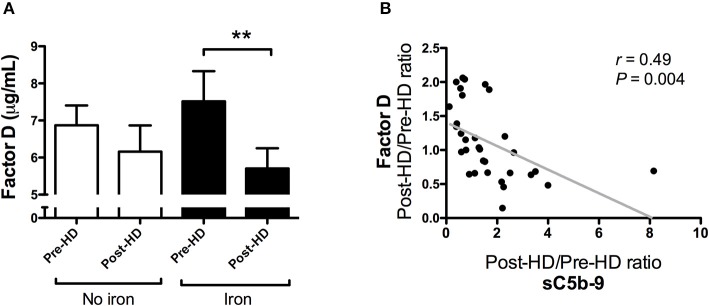
The relative decrease in factor D correlates with the relative increase of soluble C5b-9 by intravenous iron in hemodialysis patients. **(A)** Plasma levels of factor D were determined in 32 hemodialysis (HD) patients during two consecutive dialysis sessions, one with intravenous iron (Iron), and one without (No iron). During each dialysis session, factor D levels were determined at baseline and after 4 h. Data are presented as mean and SEM. **(B)** The correlation between the relative change of sC5b-9 and factor D between the two HD sessions. Correlations were evaluated using the Spearman rank correlation coefficient and paired sample *t*-test was used to compare values before and after iron infusion. *P* < 0.05 were considered to be statistically significant (^**^P < 0.01).

Once again, levels of C1q, properdin, factor D, and MBL were assessed to study the effect of IV iron on complement components (Table 3). Factor D levels did not significantly change during standard hemodialysis (*P* = 0.39, Figure 5A), but were significantly reduced after the dialysis session with IV iron infusion (*P* = 0.002; Figure 5A). Moreover, the relative decrease of factor D by IV iron correlated with the relative increase of sC5b-9 by IV iron (*r* = 0.49, *P* = 0.004; Figure 5B), suggesting that consumption of factor D is linked to complement activation. Administration of IV iron during dialysis did not significantly affect MBL, C1q or properdin levels (Table 3).

**Table 3:** The effect of intravenous iron on complement levels in dialysis patients and the correlation with soluble C5b-9.

	**No iron**			**Iron**			**sC5b-9**	
	**Pre-HD**	**Post-HD**	P^*^	**Pre-HD**	**Post-HD**	***P^%^***	***R***	***P***
**C1q** (μg/mL)	39.7 ± 9.8	43.2 ± 10.0	0.6	42.06 ± 10.3	40.8 ± 9.3	0.8	−0.13	0.4
**MBL**(ng/mL)	862 ± 145	*1, 087*±229	0.1	*1, 660*±688	*1, 518*±570	0.8	0.03	0.9
**Properdin** (μg/mL)	9.1 ± 1.2	9.6 ± 6.3	0.8	11.4 ± 1.4	12.0 ± 1.8	0.6	−0.30	0.4

Values are expressed as mean ± standard error of the mean. P^*^ indicate P-values for the difference between samples pre and post-HD in the HD session without iron tested by paired t-test. P^%^ indicate P-values for the difference between samples pre and post-HD in the HD session with iron tested by paired t-test. In the last column, R indicates Pearson correlation coefficient between Post-HD/Pre-HD ratio between the sessions of the complement component compared with the Post-HD/Pre-HD ratio between the sessions of sC5b-9.

### Intravenous Iron Sucrose Leads to Higher MPO Levels in Hemodialysis Patients, and Is Correlated With Complement Activation

**Figure 6:**
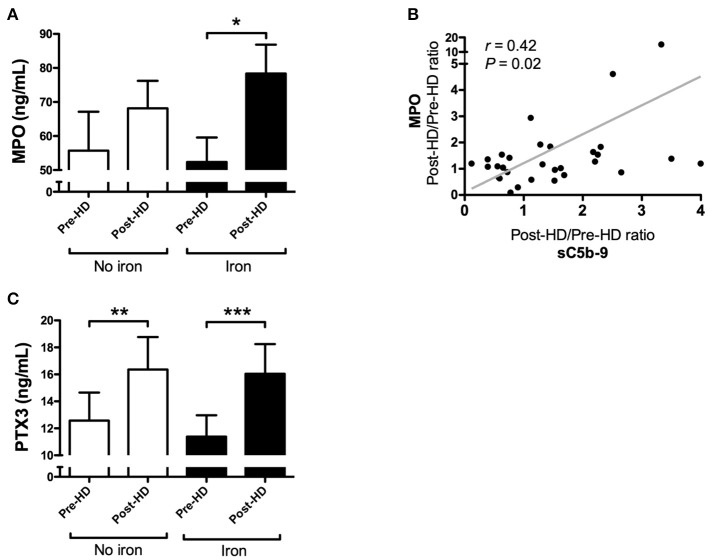
Myeloperoxidase correlates with the relative increase of soluble C5b-9 in the dialysis session with iron, whereas pentraxin-3 increases in both dialysis sessions. **(A)** Plasma levels of myeloperoxidase (MPO) were determined in 32 hemodialysis (HD) patients during two consecutive dialysis sessions, one with intravenous iron (Iron), and one without (No iron). During each dialysis session, MPO levels were determined at baseline and after 4 h. Data are presented as mean and SEM. **(B)** The correlation between the relative change of sC5b-9 and MPO between the two HD sessions. Correlations were evaluated using the Spearman rank correlation coefficient and paired sample *t*-test was used to compare values before and after iron infusion. *P* < 0.05 were considered to be statistically significant. **(C)** Furthermore, plasma levels of pentraxin-3 (PTX3) were determined in 32 hemodialysis patients during two consecutive dialysis sessions, one with intravenous iron (Iron), and one without (No iron). During each dialysis session, PTX3 levels were determined at baseline and after 4 h. Data are presented as mean and SEM. The paired sample *t*-test was used to compare values before and after iron infusion. *P*-values <0.05 were considered to be statistically significant (^*^P < 0.05, ^**^P < 0.01, ^***^P < 0.001).

The dialysis session without IV iron infusion showed no significant changes in MPO levels (*P* = 0.36; Figure 6A), while, the dialysis session with IV iron resulted in a significant rise in MPO levels (*P* = 0.02; Figure 6A). Levels of MPO showed an increase of 47% compared to baseline. Moreover, the relative increase of MPO levels by IV iron correlated significantly with the relative increase in C5b-9 levels by IV iron (*r* = 0.42, *P* = 0.02; Figure 6B). Levels of PTX3 were already significantly increased by dialysis itself (*P* = 0.002; Figure 6C) and administration of IV iron did not result in a further increase.

## Discussion

The current study demonstrates, for the first time, that administration of iron sucrose, but not ferric carboxymaltose, results in complement activation *in-vivo*. Iron sucrose induced complement activation in both non-dialysis and dialysis patients. However, while in the majority of patients iron sucrose led to complement activation, the magnitude of the response to IV iron varied considerably among the patients. Complement activation induced by iron sucrose is possibly mediated via the AP, since factor D consumption by IV iron correlated with increased levels of sC5b-9. However, involvement of the LP cannot be excluded since iron infusion in the non-dialysis patients resulted in lower MBL levels. Finally, iron sucrose significantly increased MPO levels during dialysis and the relative increase of MPO correlated with complement activation. This finding suggests that complement activation induced by IV-iron is linked to neutrophil activation resulting in oxidative stress.

Previously, our group tested the *in-vitro* ability of different iron formulations to activate the complement system (8). Based on the *in-vitro* studies, complement activation by iron sucrose was thought to occur via LP activation. The current findings implicate AP and possibly LP activation by iron sucrose. We found a significant reduction of factor D by IV iron and also a trend for lower properdin in the non-dialysis group, implying AP involvement. The significant decrease in MBL levels in the non-dialysis group could suggest a role for LP activation by iron sucrose. Recent studies indicate that activation of the AP can be mediated via the LP, more specifically initiation of the MBL-MASP complex can result in the conversion of pro-factor D in factor D by MASP-3 (11). The mechanisms behind complement activation induced by iron drugs are still unclear. A crucial difference between the iron formulations are the carbohydrate ligands which impacts the immunoreactivity as well as the stability of the molecule and the release of iron (12). In our study, iron sucrose was shown to be a complement activator while ferric carboxymaltose was not. In conformity, Fell et al. demonstrated that iron sucrose was the only formulation to induce monocyte activation (13). We propose that low stability and high labile iron release are major determinants for complement activation by IV iron. Therefore, we would expect that of the iron drugs that were not tested yet, low-molecular weight iron dextran and ferric gluconate lead to complement activation, but not ferumoxytol and iron isomaltoside.

The use of IV iron led to complement activation in the majority of the patients, however, the individual response to iron varied per patient. At present, it is not clear which determinants affect the degree of complement activation. Based on our findings, the role of the underlying cause of anemia seems limited, since complement activation was seen in non-CKD, ND-CKD, and dialysis patients. Furthermore, the rate of infusion has been previously shown to be crucial for both, hypersensitivity reactions and complement activation (14–16). However, considering current infusion protocols and newer iron formulations, the administration rate cannot explain the complement activation by IV iron seen in this study. In addition, the dose of iron could also affect the extent of complement activation. We did not see a dose-dependent effect of iron sucrose on complement (data not shown). However, our subgroups were small and therefore our conclusions are limited. Finally, factors such as genetic predisposition and patient characteristics, e.g., age and sex, might also impact iron-induced complement activation (17).

Clinically, complement activation can lead to acute and chronic effects. Acute complement activation by IV iron could lead to CARPA, a hypersensitivity reaction that is complement mediated (5). In the current study no hypersensitivity reactions occurred, which is compatible with the fact that we did not see a 5-10-fold increase in sC5b-9, which is necessary for the development of CARPA (16). Therefore, our studies only function as a proof of principle that IV iron can induce complement activation *in-vivo*, but do not prove nor exclude the concept of CARPA. Moreover, chronic complement activation by IV Iron could contribute to prolonged/long-term oxidative stress and inflammation as well as lead to complement consumption thereby increasing infection risk. Although a recent study by Macdougall et al. demonstrated the superiority of a high-dose iron-sucrose regimen compared to a low-dose regimen, limitations from their study design should be taken into account (4). First, no control group without iron administration was included, for obvious reasons. Second, regimen with other iron formulations were not compared. As such, the safety about long-term IV iron administration, particularly iron sucrose, is still moot.

MPO is a protein primarily released by activated neutrophils (18). MPO has been considered an important pathophysiological factor in oxidative stress, contributing to the activation of pro-atherogenic and inflammatory pathways, and to a higher cardiovascular risk in CKD patients (19, 20). In addition, MPO release has also been linked to iron infusion in an animal model (21). We therefore selected MPO as a potential marker of IV iron induced oxidative stress. Indeed, our results show that iron administration can contribute to increased levels of MPO, most likely through complement activation. We speculate that iron-induced complement activation leads to neutrophil activation resulting in the increased secretion of MPO, as shown by March et al., and this could potentially serve as a positive feedback loop for further complement activation (22–25). The significant increase in MPO levels was only seen in the context of dialysis. Possibly, the inflammatory environment caused by the dialysis procedure primed the neutrophils, subsequently making them more prone to complement activation-mediated oxidative stress, as supported by earlier studies (26–28).

Previously, Malindretos et al. studied the effect of slow infused IV iron on inflammation by measuring IL-6, CRP, and TNF-α (29). In their study, HD itself resulted in an increase of inflammatory parameters, while IV iron did not lead to further increase in these markers (29). Therefore, we proceeded to investigate iron-induced inflammation by using a different marker, namely PTX3 which was previously shown to be a sensitive early marker of inflammation induced by HD (30–32). Although our results confirmed the increase of PTX3 levels during HD, there was no further increase with iron administration, nor a correlation with complement activation. Possibly, the inflammatory environment of HD blurs the effect of iron on PTX3.

The strengths of our study include the use of two distinct populations (dialysis and non-dialysis) and the size of our groups. In the dialysis group, an important detail of our study design is the comparison of two different dialysis sessions in the same patient, thereby controlling for the inter-individual response to dialysis and to iron (33, 34). In addition, the panel of complement measurements that were involved in the present study permitted insight in the complement pathways that leads to iron-induced complement activation. Limitations of our study include the use of only two available iron preparations. Although we compared two of the most commonly used iron formulations, not all available iron formulations were investigated. Testing low molecular weight iron dextran would be especially interesting since it gave the strongest complement activation in previous *in-vitro* analysis. Furthermore, we did not test different infusion rates and samples were collected only 1 h after IV iron. Collecting samples at other time points would give us more detailed information. Lastly, the results in the current study are associations and therefore causality cannot be proven. Unfortunately, there was no follow up and therefore we cannot evaluate long-term effects of IV iron.

In conclusion, IV iron sucrose leads to complement activation *in-vivo*, which partially mediates iron-induced oxidative stress. Our findings suggests that complement activation by IV iron *in-vivo* possibly occurs via AP, although a contribution of the LP cannot be excluded. However, the mechanisms through which iron can activate the complement system remain limited and warrant further research. Lastly, inflammation induced by IV iron is not related to PTX3 and therefore other inflammatory markers should be investigated.

## Data Availability

All datasets generated for this study are included in the manuscript and/or the supplementary files.

## Ethics Statement

The study was approved by the local ethical committee and performed according to the principles of the declaration of Helsinki. All participants gave informed consent.

## Author Contributions

BF, MG, FP, MD, and MS: research idea and study design. BF, MG, and FP: data acquisition. BF, MG, FP, CF, MP, SB, MD, CG, and MS: data analysis and interpretation. BF, MG, and FP: statistical analysis and wrote the manuscript. All authors were involved in editing, read, and approved the final manuscript.

### Conflict of Interest Statement

CG received speaking fees and research funding from Vifor Pharma. The remaining authors declare that the research was conducted in the absence of any commercial or financial relationships that could be construed as a potential conflict of interest.

## Abbreviations

AP: Alternative pathway
CARPA: Complement activation related pseudoallergy
CKD: Chronic kidney disease
ND-CKD: Non-dialysis chronic kidney disease
CP: Classical pathway
CRP: C reactive protein
EDTA: Ethylenediaminetetraacetic acid
HD: Hemodialysis
IL-6: Interleukin-6
IV: Intravenous
LP: Lectin pathway
MAC: Membrane attack complex
MBL: Mannose-binding lectin
MPO: Myeloperoxidase
PTX3: Pentraxin-3
sC5b-9: soluble membrane attack complex
SEM: Standard error of the mean
TNF-α: Tumor necrosis factor alpha.
